# Impact of the Affordable Care Act on Disparities in Access to and Outcomes After Coronary Artery Bypass Grafting

**DOI:** 10.1007/s40615-022-01455-8

**Published:** 2022-11-16

**Authors:** Marisa Hernandez-Morgan, Christine Stypula, Matthew Fischer, Tristan Grogan, Jacques Neelankavil

**Affiliations:** Division of Cardiac Anesthesiology, Los Angeles, Department of Anesthesiology and Perioperative Medicine, University of California, Los Angeles, CA USA

**Keywords:** Affordable care act impact, Racial disparities, Cardiac surgery, CABG outcome disparities, CABG access

## Abstract

**Objectives:**

The aim of this study was to examine the effect of implementation of the Affordable Care Act’s Medicaid expansion on access to and outcomes after coronary artery bypass grafting (CABG) surgery.

**Methods:**

Retrospective observational study utilizing the Healthcare Cost and Utilization Project (HCUP) National Inpatient Sample (NIS) from 2011 to 2016. The southern region of the USA was used as a control and the western region as the implementation group. Univariate regression models and interrupted time series models were created to evaluate and assess the impact of the Affordable Care Act’s Medicaid expansion on mortality after CABG with respect to patient race.

**Results:**

From 2011 to 2016, a total of 117,819 isolated CABG operations were identified in the specified regions using the HCUP NIS. Of these, 89,918 were performed in the southern region, and the remainder were performed in the western region. The proportion of African American patients with Medicaid increased significantly in the western region after the ACA Medicaid expansion, from 13.1 to 17.6%, *p* = 0.034. There was no significant increase seen in the number of African American patients with Medicaid in the southern region. We found that overall, Black patients had higher mortality after CABG as compared to white patients (OR 1.15, *p* = 0.02); however, when broken down by region we found higher mortality among African American patients in the southern region only, with no statistically significant difference in mortality between white and Black patients in the western region.

**Conclusions:**

Implementation of the Affordable Care Act increased access to Medicaid among Black Americans but did not necessarily decrease the disparity in access to CABG or mortality after CABG between Black and white patients. When it comes to racial disparities in mortality after CABG, there are significant regional and geographic variations which have not been previously described. This finding has important implications for the development of policy and other strategies that aim to reduce these disparities.

**Supplementary Information:**

The online version contains supplementary material available at 10.1007/s40615-022-01455-8.

## Introduction

Black Americans have a higher mortality rate after coronary artery bypass grafting (CABG) than their white counterparts [1–4]. In addition, they have higher 30-day readmission rates, postoperative complications, and longer lengths of stay following CABG than other matched populations. Hypotheses include that Black Americans suffer from higher rates of chronic diseases such as hypertension and diabetes, which have been shown to increase the risk for poor postoperative outcomes [5]. While the reason for this disparity in operative mortality is likely multifactorial, structural determinants of health, including income, education, and access to healthcare, are certain to play a key role [6].

The Affordable Care Act (ACA) Medicaid Expansion went into effect between January 1, 2014 and January 1, 2015 in twenty-seven states and the District of Columbia, while twenty-three states did not expand access to Medicaid. Implementation of the ACA may result in improved access to preventative as well as surgical services and potentially improved outcomes after surgery [7], though evidence to date looking at the impact of the ACA Medicaid Expansion on surgical outcomes is sparse. The majority of states in the southern region of the USA did not use the Affordable Care Act to expand access to Medicaid while a majority of states in the western region of the country did. This clear geographic separation provides the basis for our analysis of the effect of implementation of the ACA Medicaid Expansion on access to and outcomes after CABG. The southern region serves as our control group given the near universal lack of Medicaid expansion, and the western region serves as the intervention group. We hypothesized that adoption of the ACA Medicaid Expansion would be associated with decreased mortality after CABG in the Black American population. While many studies have examined the lack of access to CABG among Black Americans, to our knowledge this is the first study to examine this disparity with respect to the time periods before and after implementation of the Affordable Care Act.

## Methods

This retrospective observational study was deemed exempt from obtaining informed consent by the affiliated institutional review board. We used the Healthcare Cost and Utilization Project (HCUP) National Inpatient Sample (NIS) to identify all hospitalizations between 2011 and 2016 in the USA in which CABG was a primary diagnosis. The NIS is an all-payor database containing discharge-level data from a 20% stratified sample of discharges from hospitals in the USA. Using United States Census Region designations, we selected only CABG procedures performed in the southern (region 3) or western (region 4) regions. There are 16 states in the southern region, only 5 states chose to implement the Medicaid expansion in 2014, the remaining 11 states did not. The western region contains 11 states, of which 7 states chose to implement the Medicaid expansion in 2014 and the remaining 4 did not. To justify our methodology, we used 2010 census data to estimate the percent of the population in each region that either did or did not implement the Medicaid expansion. By population, the percentage of states in the western region that expanded Medicaid was 92% of the region’s population. Similarly in the southern region, states that did not expand Medicaid represent 86% of the population of the region. Given these differences, the southern region served as our control group and the western region as the implementation group. For the time period from 2011 to October 2015, we identified CABG procedures using procedure and diagnostic codes from the International Classification of Diseases, Ninth Review, Clinical Modification. For the time period from October 2015 through 2016 we used procedure and diagnostic codes from the International Classification of Diseases, Tenth Review. Inclusion criteria included a primary procedure diagnosis of CABG with ICD-9 codes 36.1–36.19 OR ICD-10 codes listed in Appendix. Patients were included if they underwent CABG during the index hospitalization. Exclusion criteria included age less than 18, or any concomitant cardiac surgery in addition to CABG. The years from 2011 to 2013 were designated the pre-ACA time period which refers to the period prior to adoption and implementation of the Affordable Care Act’s Medicaid expansion. The years 2014–2016 refer to the post-ACA period. Patient demographic information including age, race, primary payor, median household income quartile for zip code, and Elixhauser Comorbidity index were gathered for each patient. Elixhauser Cormorbidity index is a method for measuring patient comorbidity based on ICD-9-CM and ICD-10 diagnosis codes gathered from hospital discharge records [8]. In addition, hospital characteristics for each patient’s hospitalization including hospital bed size, teaching status, and hospital region (urban vs. rural) were evaluated. Our primary outcome variable of interest was inpatient mortality. Additionally, we looked at hospital length of stay as a secondary outcome.

### Statistical Analysis

Patient demographics and characteristics were summarized by geographical region for the pre and post-ACA time periods using medians (IQR) or frequencies (%) unless otherwise noted, and formally compared using the Wilcoxon rank test and *t* test for continuous variables and chi-squared test for categorical variables. Statistical analyses were performed using IBM SPSS V28 *(Armonk, NY) and* R V4.1.0 *(*www.r-project.org*, Vienna, AU)*, and *p* values < 0.05 were considered statistically significant.

Univariate analysis was performed to evaluate the proportion of African American patients with Medicaid in each region for the pre and post-ACA time periods. We examined the change in proportion of African American patients receiving Medicaid who underwent CABG during the study period by constructing interrupted time series models for both the southern and western regions. We then created univariate regression models for our outcomes of interest (logistic regression for mortality and linear regression for length of stay) in order to identify variables that were significantly associated with the outcome variable, while controlling for geographic region. From our univariate regression models, we identified seven variables associated with mortality which were: race, age, patient primary payor status, patient zip code income quartile, Elixhauser comorbidity index, and hospital control. We identified nine variables associated with hospital length of stay which included those listed above in addition to hospital size and hospital location and teaching status. To evaluate the change in our outcomes of interest over time, we constructed interrupted time series models for each region and adjusted for the variables listed above.

## Results

### Study Population Characteristics

From 2011 to 2016, a total of 117,819 isolated CABG operations were identified in the specified regions using the HCUP NIS. Of these, 89,918 were performed in the southern region (region 3) with the remainder performed in the western region (region 4) (Table 1). The total number of CABG procedures performed in each region over time can be seen in Table 2. Patients who underwent CABG in the southern region were more often male, white (89%), with a mean age of 65. A majority of them had Medicare or private insurance and underwent CABG at large urban teaching hospitals. There was little change in these demographics over time with the exception that more patients were seen in urban teaching hospitals and fewer in urban non-teaching hospitals.

**Table 1:** Study sample demographics by region

	Region 3 (southern states)			Region 4 (western states)		
	Pre-ACA (*n* = 46,065)	Post-ACA (*n* = 44,289)	*p* value	Pre-ACA (14,638)	Post-ACA (13,504)	*p* value
Mortality	1142 (2.5%)	1113 (2.5%)	0.726	385 (2.6%)	340 (2.5%)	0.561
Age, mean (SD)	65.5 (10.6)	65.5 (10.3)	0.321	67.9 (10.3)	67.8 (10.0)	0.561
Zip code income			< 0.001			< 0.001
Quartile 1	17,787 (39.5%)	16,989 (39.1%)		2158 (15.2%)	2578 (19.6%)	
Quartile 2	12,411 (27.5%)	12,519 (28.8%)		3344 (23.5%)	3288 (25.1%)	
Quartile 3	9296 (20.6%)	8668 (20%)		4356 (30.7%)	3912 (29.8%)	
Quartile 4	5560 (12.3%)	5259 (12.1%)		4344 (30.6%)	3343 (25.5%)	
Primary payor			< 0.001			< 0.001
Medicare	25,685 (56.0%)	24,730 (56%)		8810 (60.2%)	8163 (60.5%)	
Medicaid	1965 (4.3%)	2306 (5.2%)		643 (4.4%)	1106 (8.2%)	
Private	14,052 (30.7%)	13,702 (31%)		4257 (29.1%)	3697 (27.4%)	
self pay	2427 (5.3%)	1782 (4%)		394 (2.7%)	139 (1%)	
No charge	308 (0.7%)	235 (0.5%)		39 (0.3%)	8 (0.1%)	
Other	1397 (3.0%)	1427 (3.2%)		490 (3.3%)	389 (2.9%)	
Race			0.062			0.051
White	41,164 (89.4%)	39,406 (89.0%)		14,024 (95.8%)	12,999 (96.3%)	
Black	4901 (10.6%)	4883 (11.0%)		614 (4.2%)	505 (3.7%)	
Female sex	12,837 (27.9%)	11,838 (26.7%)	< 0.001	3568 (24.4%)	3053 (22.6%)	< 0.001
LOS	9.70 (7.16)	9.74 (7.09)	0.382	9.59 (7.44)	9.45 (6.70)	0.101
Discharge location			< 0.001			< 0.001
Routine	22,320 (48.5%)	20,159 (45.6%)		7230 (49.4%)	6102 (45.2%)	
Short-term hospital	270 (0.6%)	297 (0.7%)		156 (1.1%)	114 (0.8%)	
Other facility	8480 (18.4%)	9256 (20.9%)		3093 (21.1%)	3111 (23.1%)	
HHC	13,815 (30.0%)	13,372 (30.2%)		3740 (25.6%)	3784 (28.1%)	
AMA	26 (0.1%)	39 (0.1%)		17 (0.1%)	33 (0.2%)	
Died	1142 (2.5%)	1113 (2.5%)		385 (2.6%)	340 (2.5%)	
Elixhauser	3.95 (2.06)	4.23 (2.10)	< 0.001	4.23 (2.24)	4.47 (2.17)	< 0.001
Hospital size			< 0.001			< 0.001
Small	3300 (7.2%)	4234 (9.6%)		889 (6.1%)	1004 (7.4%)	
Med	8655 (18.8%)	13,110 (29.6%)		2844 (19.4%)	3677 (27.2%)	
Large	34,110 (74.0%)	26,945 (60.8%)		10,893 (74.5%)	8823 (65.3%)	
Hospital location			< 0.001			< 0.001
Rural	2497 (5.4%)	1642 (3.7%)		32 (0.2%)	30 (0.2%)	
Urban not teaching	17,045 (37.0%)	10,202 (23%)		8242 (56.4%)	3903 (28.9%)	
Urban teaching	26,523 (57.6%)	32,445 (73.3%)		6352 (43.4%)	9571 (70.9%)	
Hospital control			< 0.001			< 0.001
Government	5527 (12.0%)	5688 (12.8%)		1022 (7.0%)	760 (5.6%)	
Private not profit	30,740 (66.7%)	29,432 (66.5%)		11,299 (77.3%)	10,686 (79.1%)	
Private profit	9798 (21.3%)	9169 (20.7%)		2305 (15.8%)	2058 (15.2%)	

*LOS* length of stay, *HHC* home health care, *AMA* against medical advice

**Table 2:** Total CABG procedures over time by region

Year	Caucasian	African-American
*a) Southern region*		
2011	14,737	1940
2012	13,420	1492
2013	13,007	1469
2014	12,962	1547
2015	13,362	1694
2016	13,082	1642
*b) Western region*		
2011	5160	243
2012	4559	179
2013	4305	192
2014	4349	160
2015	4344	164
2016	4306	181

Patients in the western region were similarly more often male, primarily white (96%), with a mean age of 68. The majority had Medicare or private insurance and also underwent CABG at large urban teaching hospitals. As in the southern region, over time significantly more patients were seen in urban teaching hospitals and fewer in urban non-teaching hospitals. There was no change in the mortality rate from the pre-ACA time period to the post-ACA period in either the southern or the western region. The southern region saw an increase in the median length of stay after CABG from the pre to the post-ACA period, whereas the length of stay in the western region was unchanged.

### Access to Medicaid and CABG

The proportion of patients with Medicaid insurance increased in both the southern (4 to 5.2%, *p* < 0.001) and the western (4.5 to 8.2%, *p* < 0.001) regions from the pre to the post-ACA period. However, among African American patients in the southern region, the proportion with Medicaid did not change significantly (9.5 to 10%, *p* = 0.402) from the pre to the post-ACA period. In contrast, in the western region where a majority of states implemented the Medicaid expansion, among African American patients the proportion with Medicaid increased from 13.1 to 17.6%, *p* = 0.034. The proportion of white patients with Medicaid also increased in the western region from 4.1 to 7.8%, *p* < 0.001. While the percentage of African American patients with Medicaid increased in the western region after implementation of the ACA, the total number of African American patients undergoing CABG did not change and in fact went down in the post-ACA period (627 patients underwent CABG pre-ACA as compared to 505 post-ACA). The total number of CABGs performed on white patients in the western region also decreased in the post-ACA period (13,770 vs. 12,999 respectively).

### Primary and Secondary Outcomes

**Fig. 1:**
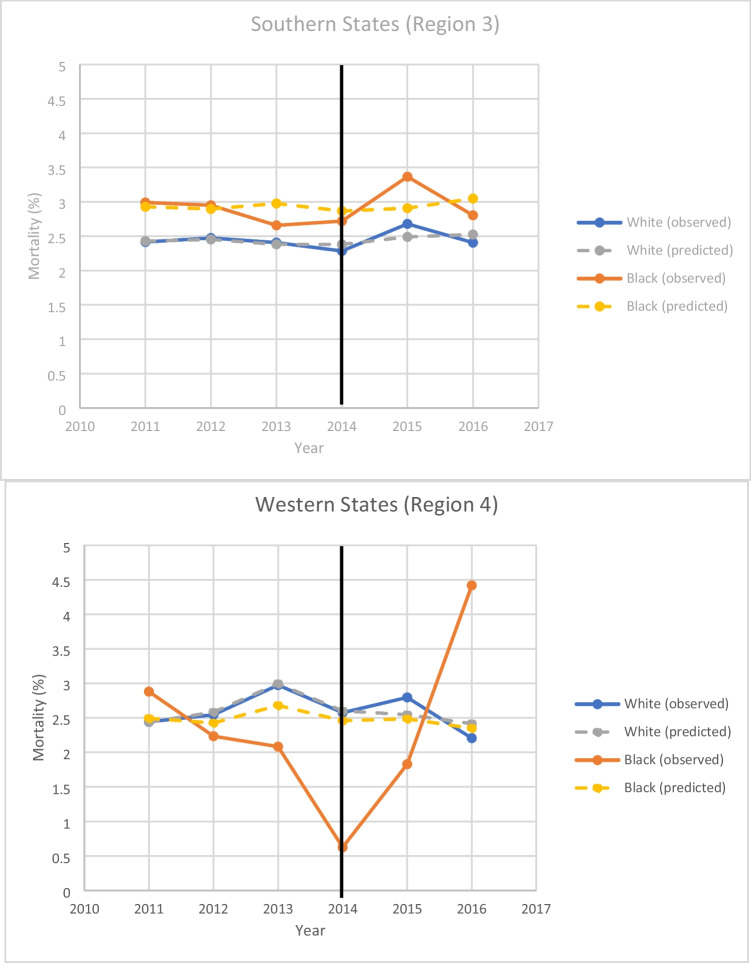
Observed and predicted mortality rate for African American and white patients by geographic region

**Fig. 2:**
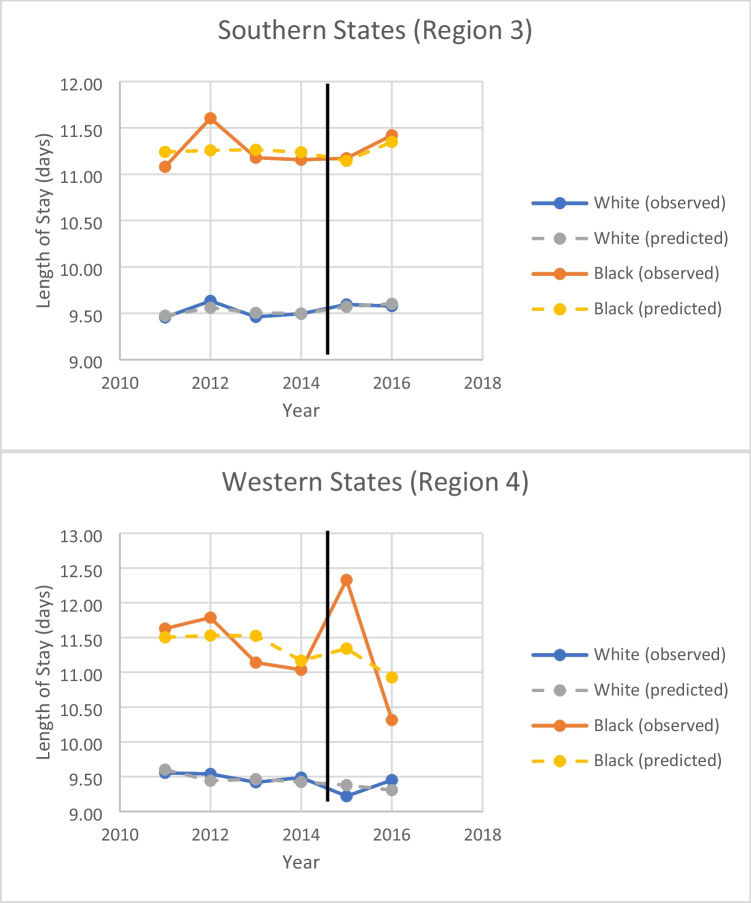
Observed and predicted hospital length of stay for African American and white patients by geographic region

On univariate analysis, Black patients had higher mortality after CABG as compared to white patients (OR 1.15, *p* = 0.02) (Tables 3 and 4) . From our interrupted time series models, we saw higher mortality among African American patients in the southern region which did not change significantly from the pre-ACA period to the post (Fig. 1). In contrast, in the western region our analysis revealed that for both the pre and the post-ACA periods, there was no statistically significant difference in mortality between white and Black patients. Length of hospital stay was significantly longer for African American patients in both regions, with no difference observed after implementation of the ACA (Fig. 2).

**Table 3:** Univariate Logistic Regression Model for Mortality Controlling for Region

	OR (95% CI)	p-value
Age Age (bootstrap)	1.52 (1.46–1.58) 1.52 (1.46–1.59)	< 0.001 < 0.001
Zip Code Quartile	0.96 (0.93–0.99)	0.018
Zip Code Quartile (bootstrap)	0.96 (0.92–0.99)	0.016
Primary Payor		< 0.001
Medicare	ref	
Medicaid	0.75 (0.63–0.89)	0.001
Private	0.50 (0.46–0.55)	< 0.001
Self-pay	0.64 (0.52–0.79)	< 0.001
No charge	0.27 (0.11–0.64)	0.003
Other	0.51 (0.40–0.66)	< 0.001
Medicaid (bootstrap)	0.75 (0.63–0.87)	0.008
Private (bootstrap)	0.50 (0.46–0.55)	0.004
Self-pay (bootstrap)	0.64( 0.51–0.77)	0.004
No charge (bootstrap)	0.27 (0.05–0.50)	0.004
Other (bootstrap)	0.51 (0.38–0.66)	0.004
African-American Race	1.17 (1.04–1.32)	0.010
African-American (bootstrap)	1.17 (1.04–1.32)	0.004
Female Sex	1.67 (1.55–1.80)	< 0.001
Female Sex (bootstrap)	1.67 (1.52–1.77)	0.004
Elixhauser Comorbidity Index	1.40 (1.38–1.43)	< 0.001
Elixhauser (bootstrap)	1.40 (1.38–1.43)	0.004
Hospital Bedsize		0.517
Small	ref	
Medium	1.07 (0.92–1.25)	0.371
Large	1.09 (0.94–1.25)	0.252
Medium (bootstrap)	1.07 (0.90–1.24)	0.410
Large (bootstrap)	1.09 (0.95–1.25)	0.307
Hospital Location		
Rural	ref	0.129
Urban Not Teaching	1.26 (1.01–1.58)	0.045
Urban Teaching	1.25 (1.00–1.56)	0.049
Urban Not Teach (bootstrap)	1.26 (1.00–1.63)	0.060
Urban Teaching (bootstrap)	1.25 (1.00–1.58)	0.048
Hospital Control		< 0.001
Government	ref	
Private Not for Profit	0.95 (0.84–1.07)	0.365
Private for Profit	1.13 (0.99–1.29)	0.074
Private Not for Profit (bootstrap)	0.95 (0.84–1.08)	0.303
Private for Profit (bootstrap)	1.13 (0.99–1.31)	0.096

**Table 4:** Univariate Logistic Regression Model for Mortality Controlling for Region (weighted)

	OR (95% CI)	p-value
Age	1.52 (1.46–1.58)	< 0.001
Zip Code Quartile	0.96 (0.92–0.99)	0.015
Primary Payor		< 0.001
Medicare	ref	
Medicaid	0.75 (0.63–0.89)	0.001
Private	0.50 (0.46–0.55)	< 0.001
Self-pay	0.64 (0.52–0.79)	< 0.001
No charge	0.27 (0.11–0.65)	0.003
Other	0.51 (0.40–0.66)	< 0.001
African-American Race	1.17 (1.04–1.32)	0.010
Female Sex	1.67 (1.55–1.80)	< 0.001
Elixhauser Comorbidity Index	1.40 (1.38–1.43)	< 0.001
Hospital Bedsize		0.497
Small	ref	
Medium	1.07 (0.92–1.25)	0.359
Large	1.09 (0.95–1.25)	0.239
Hospital Location		0.145
Rural	Ref	
Urban Not Teaching	1.25 (1.00–1.57)	0.051
Urban Teaching	1.24 (0.99–1.55)	0.056
Hospital Control		< 0.001
Government	ref	
Private Not for Profit	0.95 (0.84–1.07)	0.401
Private for Profit	1.13 (0.99–1.29)	0.074

## Discussion

We used the Healthcare Cost and Utilization Project National Inpatient Sample to examine the impact of the Affordable Care Act on access to and outcomes after CABG surgery. Our analysis showed that implementation of the Affordable Care Act increased access to Medicaid among Black Americans but did not necessarily increase access to CABG surgery or decrease the disparity in mortality between Black and white patients. We also found that when it comes to racial disparities in mortality after CABG, there are significant regional and geographic variations. This finding has important implications for the development of policy and other strategies that aim to reduce these disparities.

### Access to Care

Several prior studies have demonstrated a lack of access to CABG among Black and other minority patients in the USA [9–12]. In 2002 the American College of Cardiology Foundation and the Henry Kaiser Foundation published a summary report of racial/ethnic differences in cardiac care. The authors found that out of 23 studies with “strong” methodology, 21 of these showed that African Americans were statistically less likely what whites to undergo CABG. In fact African Americans were about one-fourth to two-thirds as likely as their white counterparts to have CABG surgery [13]. No study has looked at the effect of the ACA Medicaid expansion on racial disparities in CABG rates. We found that the expansion of Medicaid in the Western region of the USA resulted in a significant increase in access to insurance among African American patients as demonstrated by a near doubling of the proportion of African American patients with Medicaid in the region. However, the increased access to Medicaid did not result in an increase in access to CABG surgery, as the percentage of CABG surgeries performed on African American patients remained the same. In this region, African Americans make up approximately 5–10% of the general population, yet only 3.7–4.4% of all CABG surgery done in the region is for African American patients. The disparity is even greater in the southern region where African Americans make up approximately 30% of the population, yet we found that only 10% of all CABG procedures are done on Black patients in this region. This suggests that little has been accomplished in terms of increasing access to CABG among Black patients over the past decade. Therefore, our study confirms the persistent lack of access to CABG surgery among Black Americans, even with increased access to insurance coverage. Whether it is primarily a problem due to lack of other financial resources, lack of available providers, not being referred for surgery, or unwillingness to undergo surgery remains to be determined, but further study is needed to understand other factors that may be preventing Black patients from gaining access to CABG procedures.

As mentioned, we did find an association between implementation of the Affordable Care Act and rates of CABG among all patients with Medicaid. The percentage of patients with Medicaid who underwent CABG in the western region nearly doubled after enactment of the ACA. This finding is significant and promising as it suggests that the ACA is performing in the way it was intended by improving access to needed procedures for vulnerable patient populations who previously did not have access. While implantation of the ACA increased access to life saving surgical procedures such as CABG, we found that unfortunately there are still racial disparities in who benefitted more from this expanded insurance coverage. While the total number of Medicaid patients undergoing CABG increased after the ACA, we found that the proportion of patients who were white actually increased more than the proportion of patients who were Black, suggesting that there is still work to be done with respect to understanding why even with increased access to insurance, Black patients are less likely to undergo surgical revascularization.

### Mortality After CABG

In agreement with the results of prior studies, we observed an association between race and mortality with African American patients being at higher risk of death after CABG overall in the study population. While we observed a higher mortality rate among black patients after CABG in the southern region, we did not expect to see a higher mortality rate among white patients after CABG in the western region over several years. This is most likely due to sample characteristics and the fact that the majority of patients undergoing CABG surgery in the western region were white, 96% in fact. It is difficult to make conclusions about the mortality rate among Black patients in the western region since they made up just 4% of the sample in that region. The mortality rate in the Western region among black patients remained low after implementation of the ACA, but may be rising as evidenced by a higher mortality rate in 2016 in the region. It is unclear whether this is an isolated finding for a single year or whether it represents a trend. It does highlight the need for continued study over the next few years. If in fact the mortality rate for African American patients in the Western region is truly rising that certainly warrants additional investigation.

The increased operative mortality after CABG among Black Americans has been well identified, though few studies have sought to fully delineate the reasons for it. Several authors have proposed that Black patients have higher prevalence of comorbidities, suffer from a lack of access to primary care, and are more likely to present emergently or with more severe disease [2]. We acknowledge that all of these factors likely play a role, and thus we adjusted for patient comorbidities and hospital characteristics in our models and still saw that African American patients had higher mortality after CABG. We did not find that increasing insurance coverage reduced the mortality disparity, though mainly because in the western region there was no significant mortality disparity to begin with. It is also possible that 2 years is simply not enough time to see the full effect of expanded insurance coverage, and if we were to examine mortality rates after CABG again in a few years we may actually see the ACA have a significant impact both on access and mortality. On the other hand, increasing access to insurance coverage may never decrease the disparity in mortality after CABG if it does not ensure that Black patients will have access to the same quality of care as their white counterparts. Multiple prior studies showed that Black patients are more likely to present to hospitals with lower CABG volume and higher than expected operative mortality. Rangrass et. al. confirmed that hospital quality contributes to the racial disparity in CABG outcomes [4]. Furthermore, Chaudhary and colleagues found that among veteran patients with TRICARE insurance and equal-access care, racial disparities in CABG outcomes were not present [14]. Thus, improving insurance coverage among Black patients will only decrease the disparity in outcomes if it results in Black patients having access to the same level and quality of care as white patients.

### Strengths and Future Directions

Our study differs from previous studies in that none of these prior studies looked for geographic variation of these disparities, nor did they assess for changes prior to and after implantation of the Affordable Care Act. When we examined the western and the southern region of the USA separately, we found a significant difference in the mortality rates of African American patients, with patients in the southern region being at much higher risk of dying after CABG. This was true both before and after implementation of the Affordable Care Act.

This significant regional variation may be related to differences in patient characteristics or differences in treatment by physicians. We did adjust for patient comorbidities; however, it is possible that our adjustment failed to capture the fact that African American patients in the southern region may be at higher risk for mortality after CABG due to other factors such as underlying frailty, poor health literacy, delay in presentation, mistrust of medical providers, etc. In order to decrease these disparities in mortality, it is crucial to better understand the underlying factors that contribute to them. Our study highlights the need for additional research done on a more local and regional level that seeks to identify not only the most significant factors contributing to disparities in access to CABG surgery, but also identifies successful strategies and best practices that have been shown to decrease disparities. Identification of regions that are making strides in decreasing disparities and the strategies they have used is a key step in working to develop and implement targeted policies that will increase health equity on a national level.

### Limitations

Our study has a number of limitations. First, while the vast majority of states in the western region were early adopters of the Affordable Care Act, four states in the region had not adopted and implemented the Medicaid expansion by 2014. Similarly, the majority of states in the southern region did not adopt Medicaid expansion; however, six states in the region did. This introduces some confounding into our results, and it is possible that there was a larger effect of the ACA on CABG outcome disparities which our analysis failed to capture because the effect was diluted by the states in the western region that did not expand Medicaid. We used 2010 US census data of state populations to attempt to quantify this dilutional effect. By population, the percentage of states in the western region that expanded Medicaid was 92% of the population in the entire region. Similarly in the southern region, states that did not expand Medicaid represent 86% of the population of the region. Therefore, while there is some dilutional effect, it is likely quite small. HCUP data represents a large national sample of CABG admissions; however, we are limited to the variables available in the dataset and recognize that several data elements that may help answer our primary question are not present in the dataset. For example, factors such as disease severity, long-term mortality, and other significant postoperative outcomes. Despite these limitations, our study represents the first evaluation of the effect of implementation of the affordable care act on racial disparities in access to and mortality after CABG.

## Conclusion

Black patients in the USA have higher mortality after coronary bypass artery grafting. This disparity remains after adjusting for patient comorbidities, income level, and hospital size. The implementation of the Affordable Care Act appears to have increased access to Medicaid for African American patients, however as of yet has not had any substantial impact on access to CABG surgery or the mortality disparity. Furthermore, there is significant regional variation when it comes to operative risk for Black patients undergoing CABG. Future research aimed at understanding the reasons for such significant geographic variation is needed.

### Supplementary Information

Below is the link to the electronic supplementary material.

Supplementary file1 (DOCX 14 KB)
