# Supplementary material for: Impact of the Affordable Care Act on Disparities in Access to and Outcomes After Coronary Artery Bypass Grafting
Source: J Racial Ethn Health Disparities. 2022 Nov 16;10(6):2783–91. doi: 10.1007/s40615-022-01455-8 (PMC10645606; doi:10.1007/s40615-022-01455-8)
Supplement: Supplementary file 1 — Supplementary file1 (DOCX 14 KB) [file 40615_2022_1455_MOESM1_ESM.docx]

0210083 Bypass Coronary Artery, One Artery from Coronary Artery with Zooplastic Tissue, Open Approach

0210088 Bypass Coronary Artery, One Artery from Right Internal Mammary with Zooplastic Tissue, Open Approach

0210089 Bypass Coronary Artery, One Artery from Left Internal Mammary with Zooplastic Tissue, Open Approach

021008C Bypass Coronary Artery, One Artery from Thoracic Artery with Zooplastic Tissue, Open Approach

021008F Bypass Coronary Artery, One Artery from Abdominal Artery with Zooplastic Tissue, Open Approach

021008W Bypass Coronary Artery, One Artery from Aorta with Zooplastic Tissue, Open Approach

0210093 Bypass Coronary Artery, One Artery from Coronary Artery with Autologous Venous Tissue, Open Approach

0210098 Bypass Coronary Artery, One Artery from Right Internal Mammary with Autologous Venous Tissue, Open Approach

0210099 Bypass Coronary Artery, One Artery from Left Internal Mammary with Autologous Venous Tissue, Open Approach

021009C Bypass Coronary Artery, One Artery from Thoracic Artery with Autologous Venous Tissue, Open Approach

021009F Bypass Coronary Artery, One Artery from Abdominal Artery with Autologous Venous Tissue, Open Approach

021009W Bypass Coronary Artery, One Artery from Aorta with Autologous Venous Tissue, Open Approach

02100A3 Bypass Coronary Artery, One Artery from Coronary Artery with Autologous Arterial Tissue, Open Approach

02100A8 Bypass Coronary Artery, One Artery from Right Internal Mammary with Autologous Arterial Tissue, Open Approach

02100A9 Bypass Coronary Artery, One Artery from Left Internal Mammary with Autologous Arterial Tissue, Open Approach

02100AC Bypass Coronary Artery, One Artery from Thoracic Artery with Autologous Arterial Tissue, Open Approach

02100AF Bypass Coronary Artery, One Artery from Abdominal Artery with Autologous Arterial Tissue, Open Approach

02100AW Bypass Coronary Artery, One Artery from Aorta with Autologous Arterial Tissue, Open Approach

02100J3 Bypass Coronary Artery, One Artery from Coronary Artery with Synthetic Substitute, Open Approach

02100J8 Bypass Coronary Artery, One Artery from Right Internal Mammary with Synthetic Substitute, Open Approach

02100J9 Bypass Coronary Artery, One Artery from Left Internal Mammary with Synthetic Substitute, Open Approach

02100JC Bypass Coronary Artery, One Artery from Thoracic Artery with Synthetic Substitute, Open Approach

02100JF Bypass Coronary Artery, One Artery from Abdominal Artery with Synthetic Substitute, Open Approach

02100JW Bypass Coronary Artery, One Artery from Aorta with Synthetic Substitute, Open Approach

02100K3 Bypass Coronary Artery, One Artery from Coronary Artery with Nonautologous Tissue Substitute, Open Approach

02100K8 Bypass Coronary Artery, One Artery from Right Internal Mammary with Nonautologous Tissue Substitute, Open Approach

02100K9 Bypass Coronary Artery, One Artery from Left Internal Mammary with Nonautologous Tissue Substitute, Open Approach

02100KC Bypass Coronary Artery, One Artery from Thoracic Artery with Nonautologous Tissue Substitute, Open Approach

02100KF Bypass Coronary Artery, One Artery from Abdominal Artery with Nonautologous Tissue Substitute, Open Approach

02100KW Bypass Coronary Artery, One Artery from Aorta with Nonautologous Tissue Substitute, Open Approach

02100Z3 Bypass Coronary Artery, One Artery from Coronary Artery, Open Approach

02100Z8 Bypass Coronary Artery, One Artery from Right Internal Mammary, Open Approach

02100Z9 Bypass Coronary Artery, One Artery from Left Internal Mammary, Open Approach

02100ZC Bypass Coronary Artery, One Artery from Thoracic Artery, Open Approach

02100ZF Bypass Coronary Artery, One Artery from Abdominal Artery, Open Approach

0210344 Bypass Coronary Artery, One Artery from Coronary Vein with Drug-eluting Intraluminal Device, Percutaneous Approach

02103D4 Bypass Coronary Artery, One Artery from Coronary Vein with Intraluminal Device, Percutaneous Approach

0210444 Bypass Coronary Artery, One Artery from Coronary Vein with Drug-eluting Intraluminal Device, Percutaneous Endoscopic Approach

0210483 Bypass Coronary Artery, One Artery from Coronary Artery with Zooplastic Tissue, Percutaneous Endoscopic Approach

0210488 Bypass Coronary Artery, One Artery from Right Internal Mammary with Zooplastic Tissue, Percutaneous Endoscopic Approach

0210489 Bypass Coronary Artery, One Artery from Left Internal Mammary with Zooplastic Tissue, Percutaneous Endoscopic Approach

021048C Bypass Coronary Artery, One Artery from Thoracic Artery with Zooplastic Tissue, Percutaneous Endoscopic Approach

021048F Bypass Coronary Artery, One Artery from Abdominal Artery with Zooplastic Tissue, Percutaneous Endoscopic Approach

021048W Bypass Coronary Artery, One Artery from Aorta with Zooplastic Tissue, Percutaneous Endoscopic Approach

0210493 Bypass Coronary Artery, One Artery from Coronary Artery with Autologous Venous Tissue, Percutaneous Endoscopic Approach

0210498 Bypass Coronary Artery, One Artery from Right Internal Mammary with Autologous Venous Tissue, Percutaneous Endoscopic Approach

0210499 Bypass Coronary Artery, One Artery from Left Internal Mammary with Autologous Venous Tissue, Percutaneous Endoscopic Approach

021049C Bypass Coronary Artery, One Artery from Thoracic Artery with Autologous Venous Tissue, Percutaneous Endoscopic Approach

021049F Bypass Coronary Artery, One Artery from Abdominal Artery with Autologous Venous Tissue, Percutaneous Endoscopic Approach

021049W Bypass Coronary Artery, One Artery from Aorta with Autologous Venous Tissue, Percutaneous Endoscopic Approach

02104A3 Bypass Coronary Artery, One Artery from Coronary Artery with Autologous Arterial Tissue, Percutaneous Endoscopic Approach

02104A8 Bypass Coronary Artery, One Artery from Right Internal Mammary with Autologous Arterial Tissue, Percutaneous Endoscopic Approach

02104A9 Bypass Coronary Artery, One Artery from Left Internal Mammary with Autologous Arterial Tissue, Percutaneous Endoscopic Approach

02104AC Bypass Coronary Artery, One Artery from Thoracic Artery with Autologous Arterial Tissue, Percutaneous Endoscopic Approach

02104AF Bypass Coronary Artery, One Artery from Abdominal Artery with Autologous Arterial Tissue, Percutaneous Endoscopic Approach

02104AW Bypass Coronary Artery, One Artery from Aorta with Autologous Arterial Tissue, Percutaneous Endoscopic Approach

02104D4 Bypass Coronary Artery, One Artery from Coronary Vein with Intraluminal Device, Percutaneous Endoscopic Approach

02104J3 Bypass Coronary Artery, One Artery from Coronary Artery with Synthetic Substitute, Percutaneous Endoscopic Approach

02104J8 Bypass Coronary Artery, One Artery from Right Internal Mammary with Synthetic Substitute, Percutaneous Endoscopic Approach

02104J9 Bypass Coronary Artery, One Artery from Left Internal Mammary with Synthetic Substitute, Percutaneous Endoscopic Approach

02104JC Bypass Coronary Artery, One Artery from Thoracic Artery with Synthetic Substitute, Percutaneous Endoscopic Approach

02104JF Bypass Coronary Artery, One Artery from Abdominal Artery with Synthetic Substitute, Percutaneous Endoscopic Approach

02104JW Bypass Coronary Artery, One Artery from Aorta with Synthetic Substitute, Percutaneous Endoscopic Approach

02104K3 Bypass Coronary Artery, One Artery from Coronary Artery with Nonautologous Tissue Substitute, Percutaneous Endoscopic Approach

02104K8 Bypass Coronary Artery, One Artery from Right Internal Mammary with Nonautologous Tissue Substitute, Percutaneous Endoscopic Approach

02104K9 Bypass Coronary Artery, One Artery from Left Internal Mammary with Nonautologous Tissue Substitute, Percutaneous Endoscopic Approach

02104KC Bypass Coronary Artery, One Artery from Thoracic Artery with Nonautologous Tissue Substitute, Percutaneous Endoscopic Approach

02104KF Bypass Coronary Artery, One Artery from Abdominal Artery with Nonautologous Tissue Substitute, Percutaneous Endoscopic Approach

02104KW Bypass Coronary Artery, One Artery from Aorta with Nonautologous Tissue Substitute, Percutaneous Endoscopic Approach

02104Z3 Bypass Coronary Artery, One Artery from Coronary Artery, Percutaneous Endoscopic Approach

02104Z8 Bypass Coronary Artery, One Artery from Right Internal Mammary, Percutaneous Endoscopic Approach

02104Z9 Bypass Coronary Artery, One Artery from Left Internal Mammary, Percutaneous Endoscopic Approach

02104ZC Bypass Coronary Artery, One Artery from Thoracic Artery, Percutaneous Endoscopic Approach

02104ZF Bypass Coronary Artery, One Artery from Abdominal Artery, Percutaneous Endoscopic Approach

0211083 Bypass Coronary Artery, Two Arteries from Coronary Artery with Zooplastic Tissue, Open Approach

0211088 Bypass Coronary Artery, Two Arteries from Right Internal Mammary with Zooplastic Tissue, Open Approach

0211089 Bypass Coronary Artery, Two Arteries from Left Internal Mammary with Zooplastic Tissue, Open Approach

021108C Bypass Coronary Artery, Two Arteries from Thoracic Artery with Zooplastic Tissue, Open Approach

021108F Bypass Coronary Artery, Two Arteries from Abdominal Artery with Zooplastic Tissue, Open Approach

021108W Bypass Coronary Artery, Two Arteries from Aorta with Zooplastic Tissue, Open Approach

0211093 Bypass Coronary Artery, Two Arteries from Coronary Artery with Autologous Venous Tissue, Open Approach

0211098 Bypass Coronary Artery, Two Arteries from Right Internal Mammary with Autologous Venous Tissue, Open Approach

0211099 Bypass Coronary Artery, Two Arteries from Left Internal Mammary with Autologous Venous Tissue, Open Approach

021109C Bypass Coronary Artery, Two Arteries from Thoracic Artery with Autologous Venous Tissue, Open Approach

021109F Bypass Coronary Artery, Two Arteries from Abdominal Artery with Autologous Venous Tissue, Open Approach

021109W Bypass Coronary Artery, Two Arteries from Aorta with Autologous Venous Tissue, Open Approach

02110A3 Bypass Coronary Artery, Two Arteries from Coronary Artery with Autologous Arterial Tissue, Open Approach

02110A8 Bypass Coronary Artery, Two Arteries from Right Internal Mammary with Autologous Arterial Tissue, Open Approach

02110A9 Bypass Coronary Artery, Two Arteries from Left Internal Mammary with Autologous Arterial Tissue, Open Approach

02110AC Bypass Coronary Artery, Two Arteries from Thoracic Artery with Autologous Arterial Tissue, Open Approach

02110AF Bypass Coronary Artery, Two Arteries from Abdominal Artery with Autologous Arterial Tissue, Open Approach

02110AW Bypass Coronary Artery, Two Arteries from Aorta with Autologous Arterial Tissue, Open Approach

02110J3 Bypass Coronary Artery, Two Arteries from Coronary Artery with Synthetic Substitute, Open Approach

02110J8 Bypass Coronary Artery, Two Arteries from Right Internal Mammary with Synthetic Substitute, Open Approach

02110J9 Bypass Coronary Artery, Two Arteries from Left Internal Mammary with Synthetic Substitute, Open Approach

02110JC Bypass Coronary Artery, Two Arteries from Thoracic Artery with Synthetic Substitute, Open Approach

02110JF Bypass Coronary Artery, Two Arteries from Abdominal Artery with Synthetic Substitute, Open Approach

02110JW Bypass Coronary Artery, Two Arteries from Aorta with Synthetic Substitute, Open Approach

02110K3 Bypass Coronary Artery, Two Arteries from Coronary Artery with Nonautologous Tissue Substitute, Open Approach

02110K8 Bypass Coronary Artery, Two Arteries from Right Internal Mammary with Nonautologous Tissue Substitute, Open Approach

02110K9 Bypass Coronary Artery, Two Arteries from Left Internal Mammary with Nonautologous Tissue Substitute, Open Approach

02110KC Bypass Coronary Artery, Two Arteries from Thoracic Artery with Nonautologous Tissue Substitute, Open Approach

02110KF Bypass Coronary Artery, Two Arteries from Abdominal Artery with Nonautologous Tissue Substitute, Open Approach

02110KW Bypass Coronary Artery, Two Arteries from Aorta with Nonautologous Tissue Substitute, Open Approach

02110Z3 Bypass Coronary Artery, Two Arteries from Coronary Artery, Open Approach

02110Z8 Bypass Coronary Artery, Two Arteries from Right Internal Mammary, Open Approach

02110Z9 Bypass Coronary Artery, Two Arteries from Left Internal Mammary, Open Approach

02110ZC Bypass Coronary Artery, Two Arteries from Thoracic Artery, Open Approach

02110ZF Bypass Coronary Artery, Two Arteries from Abdominal Artery, Open Approach

0211344 Bypass Coronary Artery, Two Arteries from Coronary Vein with Drug-eluting Intraluminal Device, Percutaneous Approach

02113D4 Bypass Coronary Artery, Two Arteries from Coronary Vein with Intraluminal Device, Percutaneous Approach

0211444 Bypass Coronary Artery, Two Arteries from Coronary Vein with Drug-eluting Intraluminal Device, Percutaneous Endoscopic Approach

0211483 Bypass Coronary Artery, Two Arteries from Coronary Artery with Zooplastic Tissue, Percutaneous Endoscopic Approach

0211488 Bypass Coronary Artery, Two Arteries from Right Internal Mammary with Zooplastic Tissue, Percutaneous Endoscopic Approach

0211489 Bypass Coronary Artery, Two Arteries from Left Internal Mammary with Zooplastic Tissue, Percutaneous Endoscopic Approach

021148C Bypass Coronary Artery, Two Arteries from Thoracic Artery with Zooplastic Tissue, Percutaneous Endoscopic Approach

021148F Bypass Coronary Artery, Two Arteries from Abdominal Artery with Zooplastic Tissue, Percutaneous Endoscopic Approach

021148W Bypass Coronary Artery, Two Arteries from Aorta with Zooplastic Tissue, Percutaneous Endoscopic Approach

0211493 Bypass Coronary Artery, Two Arteries from Coronary Artery with Autologous Venous Tissue, Percutaneous Endoscopic Approach

0211498 Bypass Coronary Artery, Two Arteries from Right Internal Mammary with Autologous Venous Tissue, Percutaneous Endoscopic Approach

0211499 Bypass Coronary Artery, Two Arteries from Left Internal Mammary with Autologous Venous Tissue, Percutaneous Endoscopic Approach

021149C Bypass Coronary Artery, Two Arteries from Thoracic Artery with Autologous Venous Tissue, Percutaneous Endoscopic Approach

021149F Bypass Coronary Artery, Two Arteries from Abdominal Artery with Autologous Venous Tissue, Percutaneous Endoscopic Approach

021149W Bypass Coronary Artery, Two Arteries from Aorta with Autologous Venous Tissue, Percutaneous Endoscopic Approach

02114A3 Bypass Coronary Artery, Two Arteries from Coronary Artery with Autologous Arterial Tissue, Percutaneous Endoscopic Approach

02114A8 Bypass Coronary Artery, Two Arteries from Right Internal Mammary with Autologous Arterial Tissue, Percutaneous Endoscopic Approach

02114A9 Bypass Coronary Artery, Two Arteries from Left Internal Mammary with Autologous Arterial Tissue, Percutaneous Endoscopic Approach

02114AC Bypass Coronary Artery, Two Arteries from Thoracic Artery with Autologous Arterial Tissue, Percutaneous Endoscopic Approach

02114AF Bypass Coronary Artery, Two Arteries from Abdominal Artery with Autologous Arterial Tissue, Percutaneous Endoscopic Approach

02114AW Bypass Coronary Artery, Two Arteries from Aorta with Autologous Arterial Tissue, Percutaneous Endoscopic Approach

02114D4 Bypass Coronary Artery, Two Arteries from Coronary Vein with Intraluminal Device, Percutaneous Endoscopic Approach

02114J3 Bypass Coronary Artery, Two Arteries from Coronary Artery with Synthetic Substitute, Percutaneous Endoscopic Approach

02114J8 Bypass Coronary Artery, Two Arteries from Right Internal Mammary with Synthetic Substitute, Percutaneous Endoscopic Approach

02114J9 Bypass Coronary Artery, Two Arteries from Left Internal Mammary with Synthetic Substitute, Percutaneous Endoscopic Approach

02114JC Bypass Coronary Artery, Two Arteries from Thoracic Artery with Synthetic Substitute, Percutaneous Endoscopic Approach

02114JF Bypass Coronary Artery, Two Arteries from Abdominal Artery with Synthetic Substitute, Percutaneous Endoscopic Approach

02114JW Bypass Coronary Artery, Two Arteries from Aorta with Synthetic Substitute, Percutaneous Endoscopic Approach

02114K3 Bypass Coronary Artery, Two Arteries from Coronary Artery with Nonautologous Tissue Substitute, Percutaneous Endoscopic Approach

02114K8 Bypass Coronary Artery, Two Arteries from Right Internal Mammary with Nonautologous Tissue Substitute, Percutaneous Endoscopic Approach

02114K9 Bypass Coronary Artery, Two Arteries from Left Internal Mammary with Nonautologous Tissue Substitute, Percutaneous Endoscopic Approach

02114KC Bypass Coronary Artery, Two Arteries from Thoracic Artery with Nonautologous Tissue Substitute, Percutaneous Endoscopic Approach

02114KF Bypass Coronary Artery, Two Arteries from Abdominal Artery with Nonautologous Tissue Substitute, Percutaneous Endoscopic Approach

02114KW Bypass Coronary Artery, Two Arteries from Aorta with Nonautologous Tissue Substitute, Percutaneous Endoscopic Approach

02114Z3 Bypass Coronary Artery, Two Arteries from Coronary Artery, Percutaneous Endoscopic Approach

02114Z8 Bypass Coronary Artery, Two Arteries from Right Internal Mammary, Percutaneous Endoscopic Approach

02114Z9 Bypass Coronary Artery, Two Arteries from Left Internal Mammary, Percutaneous Endoscopic Approach

02114ZC Bypass Coronary Artery, Two Arteries from Thoracic Artery, Percutaneous Endoscopic Approach

02114ZF Bypass Coronary Artery, Two Arteries from Abdominal Artery, Percutaneous Endoscopic Approach

0212083 Bypass Coronary Artery, Three Arteries from Coronary Artery with Zooplastic Tissue, Open Approach

0212088 Bypass Coronary Artery, Three Arteries from Right Internal Mammary with Zooplastic Tissue, Open Approach

0212089 Bypass Coronary Artery, Three Arteries from Left Internal Mammary with Zooplastic Tissue, Open Approach

021208C Bypass Coronary Artery, Three Arteries from Thoracic Artery with Zooplastic Tissue, Open Approach

021208F Bypass Coronary Artery, Three Arteries from Abdominal Artery with Zooplastic Tissue, Open Approach

021208W Bypass Coronary Artery, Three Arteries from Aorta with Zooplastic Tissue, Open Approach

0212093 Bypass Coronary Artery, Three Arteries from Coronary Artery with Autologous Venous Tissue, Open Approach

0212098 Bypass Coronary Artery, Three Arteries from Right Internal Mammary with Autologous Venous Tissue, Open Approach

0212099 Bypass Coronary Artery, Three Arteries from Left Internal Mammary with Autologous Venous Tissue, Open Approach

021209C Bypass Coronary Artery, Three Arteries from Thoracic Artery with Autologous Venous Tissue, Open Approach

021209F Bypass Coronary Artery, Three Arteries from Abdominal Artery with Autologous Venous Tissue, Open Approach

021209W Bypass Coronary Artery, Three Arteries from Aorta with Autologous Venous Tissue, Open Approach

02120A3 Bypass Coronary Artery, Three Arteries from Coronary Artery with Autologous Arterial Tissue, Open Approach

02120A8 Bypass Coronary Artery, Three Arteries from Right Internal Mammary with Autologous Arterial Tissue, Open Approach

02120A9 Bypass Coronary Artery, Three Arteries from Left Internal Mammary with Autologous Arterial Tissue, Open Approach

02120AC Bypass Coronary Artery, Three Arteries from Thoracic Artery with Autologous Arterial Tissue, Open Approach

02120AF Bypass Coronary Artery, Three Arteries from Abdominal Artery with Autologous Arterial Tissue, Open Approach

02120AW Bypass Coronary Artery, Three Arteries from Aorta with Autologous Arterial Tissue, Open Approach

02120J3 Bypass Coronary Artery, Three Arteries from Coronary Artery with Synthetic Substitute, Open Approach

02120J8 Bypass Coronary Artery, Three Arteries from Right Internal Mammary with Synthetic Substitute, Open Approach

02120J9 Bypass Coronary Artery, Three Arteries from Left Internal Mammary with Synthetic Substitute, Open Approach

02120JC Bypass Coronary Artery, Three Arteries from Thoracic Artery with Synthetic Substitute, Open Approach

02120JF Bypass Coronary Artery, Three Arteries from Abdominal Artery with Synthetic Substitute, Open Approach

02120JW Bypass Coronary Artery, Three Arteries from Aorta with Synthetic Substitute, Open Approach

02120K3 Bypass Coronary Artery, Three Arteries from Coronary Artery with Nonautologous Tissue Substitute, Open Approach

02120K8 Bypass Coronary Artery, Three Arteries from Right Internal Mammary with Nonautologous Tissue Substitute, Open Approach

02120K9 Bypass Coronary Artery, Three Arteries from Left Internal Mammary with Nonautologous Tissue Substitute, Open Approach

02120KC Bypass Coronary Artery, Three Arteries from Thoracic Artery with Nonautologous Tissue Substitute, Open Approach

02120KF Bypass Coronary Artery, Three Arteries from Abdominal Artery with Nonautologous Tissue Substitute, Open Approach

02120KW Bypass Coronary Artery, Three Arteries from Aorta with Nonautologous Tissue Substitute, Open Approach

02120Z3 Bypass Coronary Artery, Three Arteries from Coronary Artery, Open Approach

02120Z8 Bypass Coronary Artery, Three Arteries from Right Internal Mammary, Open Approach

02120Z9 Bypass Coronary Artery, Three Arteries from Left Internal Mammary, Open Approach

02120ZC Bypass Coronary Artery, Three Arteries from Thoracic Artery, Open Approach

02120ZF Bypass Coronary Artery, Three Arteries from Abdominal Artery, Open Approach

0212344 Bypass Coronary Artery, Three Arteries from Coronary Vein with Drug-eluting Intraluminal Device, Percutaneous Approach

02123D4 Bypass Coronary Artery, Three Arteries from Coronary Vein with Intraluminal Device, Percutaneous Approach

0212444 Bypass Coronary Artery, Three Arteries from Coronary Vein with Drug-eluting Intraluminal Device, Percutaneous Endoscopic Approach

0212483 Bypass Coronary Artery, Three Arteries from Coronary Artery with Zooplastic Tissue, Percutaneous Endoscopic Approach

0212488 Bypass Coronary Artery, Three Arteries from Right Internal Mammary with Zooplastic Tissue, Percutaneous Endoscopic Approach

0212489 Bypass Coronary Artery, Three Arteries from Left Internal Mammary with Zooplastic Tissue, Percutaneous Endoscopic Approach

021248C Bypass Coronary Artery, Three Arteries from Thoracic Artery with Zooplastic Tissue, Percutaneous Endoscopic Approach

021248F Bypass Coronary Artery, Three Arteries from Abdominal Artery with Zooplastic Tissue, Percutaneous Endoscopic Approach

021248W Bypass Coronary Artery, Three Arteries from Aorta with Zooplastic Tissue, Percutaneous Endoscopic Approach

0212493 Bypass Coronary Artery, Three Arteries from Coronary Artery with Autologous Venous Tissue, Percutaneous Endoscopic Approach

0212498 Bypass Coronary Artery, Three Arteries from Right Internal Mammary with Autologous Venous Tissue, Percutaneous Endoscopic Approach

0212499 Bypass Coronary Artery, Three Arteries from Left Internal Mammary with Autologous Venous Tissue, Percutaneous Endoscopic Approach

021249C Bypass Coronary Artery, Three Arteries from Thoracic Artery with Autologous Venous Tissue, Percutaneous Endoscopic Approach

021249F Bypass Coronary Artery, Three Arteries from Abdominal Artery with Autologous Venous Tissue, Percutaneous Endoscopic Approach

021249W Bypass Coronary Artery, Three Arteries from Aorta with Autologous Venous Tissue, Percutaneous Endoscopic Approach

02124A3 Bypass Coronary Artery, Three Arteries from Coronary Artery with Autologous Arterial Tissue, Percutaneous Endoscopic Approach

02124A8 Bypass Coronary Artery, Three Arteries from Right Internal Mammary with Autologous Arterial Tissue, Percutaneous Endoscopic Approach

02124A9 Bypass Coronary Artery, Three Arteries from Left Internal Mammary with Autologous Arterial Tissue, Percutaneous Endoscopic Approach

02124AC Bypass Coronary Artery, Three Arteries from Thoracic Artery with Autologous Arterial Tissue, Percutaneous Endoscopic Approach

02124AF Bypass Coronary Artery, Three Arteries from Abdominal Artery with Autologous Arterial Tissue, Percutaneous Endoscopic Approach

02124AW Bypass Coronary Artery, Three Arteries from Aorta with Autologous Arterial Tissue, Percutaneous Endoscopic Approach

02124D4 Bypass Coronary Artery, Three Arteries from Coronary Vein with Intraluminal Device, Percutaneous Endoscopic Approach

02124J3 Bypass Coronary Artery, Three Arteries from Coronary Artery with Synthetic Substitute, Percutaneous Endoscopic Approach

02124J8 Bypass Coronary Artery, Three Arteries from Right Internal Mammary with Synthetic Substitute, Percutaneous Endoscopic Approach

02124J9 Bypass Coronary Artery, Three Arteries from Left Internal Mammary with Synthetic Substitute, Percutaneous Endoscopic Approach

02124JC Bypass Coronary Artery, Three Arteries from Thoracic Artery with Synthetic Substitute, Percutaneous Endoscopic Approach

02124JF Bypass Coronary Artery, Three Arteries from Abdominal Artery with Synthetic Substitute, Percutaneous Endoscopic Approach

02124JW Bypass Coronary Artery, Three Arteries from Aorta with Synthetic Substitute, Percutaneous Endoscopic Approach

02124K3 Bypass Coronary Artery, Three Arteries from Coronary Artery with Nonautologous Tissue Substitute, Percutaneous Endoscopic Approach

02124K8 Bypass Coronary Artery, Three Arteries from Right Internal Mammary with Nonautologous Tissue Substitute, Percutaneous Endoscopic Approach

02124K9 Bypass Coronary Artery, Three Arteries from Left Internal Mammary with Nonautologous Tissue Substitute, Percutaneous Endoscopic Approach

02124KC Bypass Coronary Artery, Three Arteries from Thoracic Artery with Nonautologous Tissue Substitute, Percutaneous Endoscopic Approach

02124KF Bypass Coronary Artery, Three Arteries from Abdominal Artery with Nonautologous Tissue Substitute, Percutaneous Endoscopic Approach

02124KW Bypass Coronary Artery, Three Arteries from Aorta with Nonautologous Tissue Substitute, Percutaneous Endoscopic Approach

02124Z3 Bypass Coronary Artery, Three Arteries from Coronary Artery, Percutaneous Endoscopic Approach

02124Z8 Bypass Coronary Artery, Three Arteries from Right Internal Mammary, Percutaneous Endoscopic Approach

02124Z9 Bypass Coronary Artery, Three Arteries from Left Internal Mammary, Percutaneous Endoscopic Approach

02124ZC Bypass Coronary Artery, Three Arteries from Thoracic Artery, Percutaneous Endoscopic Approach

02124ZF Bypass Coronary Artery, Three Arteries from Abdominal Artery, Percutaneous Endoscopic Approach

0213083 Bypass Coronary Artery, Four or More Arteries from Coronary Artery with Zooplastic Tissue, Open Approach

0213088 Bypass Coronary Artery, Four or More Arteries from Right Internal Mammary with Zooplastic Tissue, Open Approach

0213089 Bypass Coronary Artery, Four or More Arteries from Left Internal Mammary with Zooplastic Tissue, Open Approach

021308C Bypass Coronary Artery, Four or More Arteries from Thoracic Artery with Zooplastic Tissue, Open Approach

021308F Bypass Coronary Artery, Four or More Arteries from Abdominal Artery with Zooplastic Tissue, Open Approach

021308W Bypass Coronary Artery, Four or More Arteries from Aorta with Zooplastic Tissue, Open Approach

0213093 Bypass Coronary Artery, Four or More Arteries from Coronary Artery with Autologous Venous Tissue, Open Approach

0213098 Bypass Coronary Artery, Four or More Arteries from Right Internal Mammary with Autologous Venous Tissue, Open Approach

0213099 Bypass Coronary Artery, Four or More Arteries from Left Internal Mammary with Autologous Venous Tissue, Open Approach

021309C Bypass Coronary Artery, Four or More Arteries from Thoracic Artery with Autologous Venous Tissue, Open Approach

021309F Bypass Coronary Artery, Four or More Arteries from Abdominal Artery with Autologous Venous Tissue, Open Approach

021309W Bypass Coronary Artery, Four or More Arteries from Aorta with Autologous Venous Tissue, Open Approach

02130A3 Bypass Coronary Artery, Four or More Arteries from Coronary Artery with Autologous Arterial Tissue, Open Approach

02130A8 Bypass Coronary Artery, Four or More Arteries from Right Internal Mammary with Autologous Arterial Tissue, Open Approach

02130A9 Bypass Coronary Artery, Four or More Arteries from Left Internal Mammary with Autologous Arterial Tissue, Open Approach

02130AC Bypass Coronary Artery, Four or More Arteries from Thoracic Artery with Autologous Arterial Tissue, Open Approach

02130AF Bypass Coronary Artery, Four or More Arteries from Abdominal Artery with Autologous Arterial Tissue, Open Approach

02130AW Bypass Coronary Artery, Four or More Arteries from Aorta with Autologous Arterial Tissue, Open Approach

02130J3 Bypass Coronary Artery, Four or More Arteries from Coronary Artery with Synthetic Substitute, Open Approach

02130J8 Bypass Coronary Artery, Four or More Arteries from Right Internal Mammary with Synthetic Substitute, Open Approach

02130J9 Bypass Coronary Artery, Four or More Arteries from Left Internal Mammary with Synthetic Substitute, Open Approach

02130JC Bypass Coronary Artery, Four or More Arteries from Thoracic Artery with Synthetic Substitute, Open Approach

02130JF Bypass Coronary Artery, Four or More Arteries from Abdominal Artery with Synthetic Substitute, Open Approach

02130JW Bypass Coronary Artery, Four or More Arteries from Aorta with Synthetic Substitute, Open Approach

02130K3 Bypass Coronary Artery, Four or More Arteries from Coronary Artery with Nonautologous Tissue Substitute, Open Approach

02130K8 Bypass Coronary Artery, Four or More Arteries from Right Internal Mammary with Nonautologous Tissue Substitute, Open Approach

02130K9 Bypass Coronary Artery, Four or More Arteries from Left Internal Mammary with Nonautologous Tissue Substitute, Open Approach

02130KC Bypass Coronary Artery, Four or More Arteries from Thoracic Artery with Nonautologous Tissue Substitute, Open Approach

02130KF Bypass Coronary Artery, Four or More Arteries from Abdominal Artery with Nonautologous Tissue Substitute, Open Approach

02130KW Bypass Coronary Artery, Four or More Arteries from Aorta with Nonautologous Tissue Substitute, Open Approach

02130Z3 Bypass Coronary Artery, Four or More Arteries from Coronary Artery, Open Approach

02130Z8 Bypass Coronary Artery, Four or More Arteries from Right Internal Mammary, Open Approach

02130Z9 Bypass Coronary Artery, Four or More Arteries from Left Internal Mammary, Open Approach

02130ZC Bypass Coronary Artery, Four or More Arteries from Thoracic Artery, Open Approach

02130ZF Bypass Coronary Artery, Four or More Arteries from Abdominal Artery, Open Approach

0213344 Bypass Coronary Artery, Four or More Arteries from Coronary Vein with Drug-eluting Intraluminal Device, Percutaneous Approach

02133D4 Bypass Coronary Artery, Four or More Arteries from Coronary Vein with Intraluminal Device, Percutaneous Approach

0213444 Bypass Coronary Artery, Four or More Arteries from Coronary Vein with Drug-eluting Intraluminal Device, Percutaneous Endoscopic Approach

0213483 Bypass Coronary Artery, Four or More Arteries from Coronary Artery with Zooplastic Tissue, Percutaneous Endoscopic Approach

0213488 Bypass Coronary Artery, Four or More Arteries from Right Internal Mammary with Zooplastic Tissue, Percutaneous Endoscopic Approach

0213489 Bypass Coronary Artery, Four or More Arteries from Left Internal Mammary with Zooplastic Tissue, Percutaneous Endoscopic Approach

021348C Bypass Coronary Artery, Four or More Arteries from Thoracic Artery with Zooplastic Tissue, Percutaneous Endoscopic Approach

021348F Bypass Coronary Artery, Four or More Arteries from Abdominal Artery with Zooplastic Tissue, Percutaneous Endoscopic Approach

021348W Bypass Coronary Artery, Four or More Arteries from Aorta with Zooplastic Tissue, Percutaneous Endoscopic Approach

0213493 Bypass Coronary Artery, Four or More Arteries from Coronary Artery with Autologous Venous Tissue, Percutaneous Endoscopic Approach

0213498 Bypass Coronary Artery, Four or More Arteries from Right Internal Mammary with Autologous Venous Tissue, Percutaneous Endoscopic Approach

0213499 Bypass Coronary Artery, Four or More Arteries from Left Internal Mammary with Autologous Venous Tissue, Percutaneous Endoscopic Approach

021349C Bypass Coronary Artery, Four or More Arteries from Thoracic Artery with Autologous Venous Tissue, Percutaneous Endoscopic Approach

021349F Bypass Coronary Artery, Four or More Arteries from Abdominal Artery with Autologous Venous Tissue, Percutaneous Endoscopic Approach

021349W Bypass Coronary Artery, Four or More Arteries from Aorta with Autologous Venous Tissue, Percutaneous Endoscopic Approach

02134A3 Bypass Coronary Artery, Four or More Arteries from Coronary Artery with Autologous Arterial Tissue, Percutaneous Endoscopic Approach

02134A8 Bypass Coronary Artery, Four or More Arteries from Right Internal Mammary with Autologous Arterial Tissue, Percutaneous Endoscopic Approach

02134A9 Bypass Coronary Artery, Four or More Arteries from Left Internal Mammary with Autologous Arterial Tissue, Percutaneous Endoscopic Approach

02134AC Bypass Coronary Artery, Four or More Arteries from Thoracic Artery with Autologous Arterial Tissue, Percutaneous Endoscopic Approach

02134AF Bypass Coronary Artery, Four or More Arteries from Abdominal Artery with Autologous Arterial Tissue, Percutaneous Endoscopic Approach

02134AW Bypass Coronary Artery, Four or More Arteries from Aorta with Autologous Arterial Tissue, Percutaneous Endoscopic Approach

02134D4 Bypass Coronary Artery, Four or More Arteries from Coronary Vein with Intraluminal Device, Percutaneous Endoscopic Approach

02134J3 Bypass Coronary Artery, Four or More Arteries from Coronary Artery with Synthetic Substitute, Percutaneous Endoscopic Approach

02134J8 Bypass Coronary Artery, Four or More Arteries from Right Internal Mammary with Synthetic Substitute, Percutaneous Endoscopic Approach

02134J9 Bypass Coronary Artery, Four or More Arteries from Left Internal Mammary with Synthetic Substitute, Percutaneous Endoscopic Approach

02134JC Bypass Coronary Artery, Four or More Arteries from Thoracic Artery with Synthetic Substitute, Percutaneous Endoscopic Approach

02134JF Bypass Coronary Artery, Four or More Arteries from Abdominal Artery with Synthetic Substitute, Percutaneous Endoscopic Approach

02134JW Bypass Coronary Artery, Four or More Arteries from Aorta with Synthetic Substitute, Percutaneous Endoscopic Approach

02134K3 Bypass Coronary Artery, Four or More Arteries from Coronary Artery with Nonautologous Tissue Substitute, Percutaneous Endoscopic Approach

02134K8 Bypass Coronary Artery, Four or More Arteries from Right Internal Mammary with Nonautologous Tissue Substitute, Percutaneous Endoscopic Approach

02134K9 Bypass Coronary Artery, Four or More Arteries from Left Internal Mammary with Nonautologous Tissue Substitute, Percutaneous Endoscopic Approach

02134KC Bypass Coronary Artery, Four or More Arteries from Thoracic Artery with Nonautologous Tissue Substitute, Percutaneous Endoscopic Approach

02134KF Bypass Coronary Artery, Four or More Arteries from Abdominal Artery with Nonautologous Tissue Substitute, Percutaneous Endoscopic Approach

02134KW Bypass Coronary Artery, Four or More Arteries from Aorta with Nonautologous Tissue Substitute, Percutaneous Endoscopic Approach

02134Z3 Bypass Coronary Artery, Four or More Arteries from Coronary Artery, Percutaneous Endoscopic Approach

02134Z8 Bypass Coronary Artery, Four or More Arteries from Right Internal Mammary, Percutaneous Endoscopic Approach

02134Z9 Bypass Coronary Artery, Four or More Arteries from Left Internal Mammary, Percutaneous Endoscopic Approach

02134ZC Bypass Coronary Artery, Four or More Arteries from Thoracic Artery, Percutaneous Endoscopic Approach

02134ZF
